# Proof of concept for high-dose Cannabidiol pretreatment to antagonize opioid induced persistent apnea in mice

**DOI:** 10.3389/fnins.2025.1654787

**Published:** 2025-10-08

**Authors:** Beth M. Wiese, Evgeny Bondarenko, Jack L. Feldman

**Affiliations:** Department of Neurobiology, David Geffen School of Medicine, UCLA, Los Angeles, CA, United States

**Keywords:** CBD, opioids, breathing, OIPA, OIRD

## Abstract

**Background:**

Opioid related fatalities remain a public health crisis in the US. Currently, the only way to restore breathing following an opioid induced persistent apnea is with the administration of the opioid antagonist naloxone, but it also reverses analgesia, euphoria, and induces precipitated withdrawal in opioid dependent individuals.

**Methods:**

Using whole-body plethysmography, we assessed changes in breathing frequency in awake behaving mice resulting from a single fentanyl dose (50 mg/kg i.p.) that followed i.p. pretreatment with saline, vehicle, naloxone (100 mg/kg), cannabidiol (CBD) (250 mg/kg), or CBD + naloxone. Then we assessed the delay to opioid-induced persistent apnea (OIPA) and the median lethal dose (LD_50_) of fentanyl during a continuous i.c.v. infusion of fentanyl (100 ng/min), in urethane anesthetized mice, following pretreatment with saline, vehicle, naloxone (100 mg/kg), CBD (250 mg/kg), or CBD + naloxone i.p.

**Results:**

Here we show acute pretreatment with CBD is as effective as naloxone at preventing opioid-induced respiratory depression from fentanyl in awake mice, and increasing LD_50_ of fentanyl in urethane anesthetized mice. When pre-administered together, CBD + naloxone, increased LD_50_ of fentanyl even more than CBD or naloxone alone in urethane anesthetized mice.

**Conclusion:**

CBD may be an effective preventative therapy for OIPA by increasing the time before apnea onset and potentially enhancing the efficacy of naloxone as an additional strategy to save lives.

## Introduction

Opioids activate *μ*-opioid receptors to produce analgesia, but also depress brainstem circuits that generate breathing movements that can result in persistent and fatal apnea (Del Negro et al., 2018; Ashhad and Feldman, 2020; Janczewski and Feldman, 2006). The present treatment to restore breathing after opioid-induced persistent apnea (OIPA) takes place with the administration of an opioid antagonist, commonly naloxone (NX), that restores breathing after the onset of OIPA but also reverses opioid-induced analgesia and euphoria and induces precipitated withdrawal in opioid-dependent individuals (Kang et al., 2022). The use of opioid antagonists requires that someone is present to administer it, which is, unfortunately, not always the case. While higher dose naloxone formulations and more potent opioid receptor antagonists are being developed to address the opioid epidemic driven by more potent synthetic opioids such as fentanyl: (i) they do nothing to expand the therapeutic window time for naloxone administration (Britch and Walsh, 2022), (ii) there is no data suggesting that higher dose naloxone formulations are advantageous over current practice of multiple dosing regimen of naloxone (Lemen et al., 2024), (iii) administration of high dose naloxone induces debilitating precipitated withdrawal in opioid dependent individuals, often requiring hospital monitoring (Lemen et al., 2024; Buajordet et al., 2004). Research focused on reducing respiratory depression and, most importantly, the incidence of OIPA, thereby expanding the therapeutic window for NX administration and/or decreasing the incidence of overdoses when NX is even required, would be of great benefit.

High-dose CBD has therapeutic benefits in the treatment of substance use disorders (Britch et al., 2021; Hurd et al., 2015; Iffland and Grotenhermen, 2017; Lo et al., 2023; Manini et al., 2015), pain (Mlost et al., 2020), anxiety (Britch et al., 2021; Iffland and Grotenhermen, 2017; Sideris and Doan, 2024), depression (Zanelati et al., 2010), and gastrointestinal ailments (Britch et al., 2021; Iffland and Grotenhermen, 2017; Sideris and Doan, 2024; Peng et al., 2022; Taylor et al., 2018). Additionally, CBD inhibits binding at opioid receptors (Britch et al., 2021; Manini et al., 2015; Mlost et al., 2020; Peng et al., 2022; Bosquez-Berger et al., 2023; Kathmann et al., 2006; Rodríguez-Muñoz et al., 2018) and increases the efficacy of NX (Bosquez-Berger et al., 2023). Other cannabinoids that act on CB1 and CB2 receptors appear to preserve normal breathing behavior in preclinical models of non-fatal opioid-induced respiratory depression (Wiese et al., 2021; Wiese et al., 2020). Yet, it remains unknown if the same is true for CBD.

We predicted that high-dose CBD pretreatment would attenuate fentanyl-induced respiratory depression in awake mice and increase the fentanyl LD_50_ during continuous fentanyl infusion in urethane anesthetized mice. For comparison with the effectiveness of CBD, we used NX, the standard agent for the reversal of fentanyl-induced apnea in humans, and, when used as pretreatment, reduces opioid-induced respiratory depression in mice (Varshneya et al., 2022). Following pretreatment in awake mice, CBD was as effective as NX pretreatment at mitigating fentanyl-induced depression of breathing frequency, while in anesthetized mice, CBD pretreatment significantly increased the time to OIPA onset and subsequently the LD_50_ of fentanyl, equal to the effect of NX pretreatment. Furthermore, CBD + NX pretreatment was more efficacious than either CBD or NX alone at increasing the time to OIPA onset and LD_50_ of fentanyl.

## Method

### Animals

Male C57 mice (*n* = 66) bred in-house under 12-h light/dark cycle had unrestricted access to food and water. Mice were used under protocols approved by the University of California, Los Angeles, Animal Research Committee and UCLA animal care committee (#1994-159-83). 32 mice were used for awake experiments [*n* = 6 pretreatment with Saline (Sigma), *n* = 7 pretreatment with Vehicle (10% DMSO (Sigma), 10% Tween-80 (Sigma), and 80% saline), *n* = 6 pretreatment with CBD (Cayman Chemical, catalog # 13956-29-1), *n* = 6 pretreatment with Naloxone (NX; Pfizer, 100 mg/kg), *n* = 7 pretreatment with NX + CBD (CBD + NX; 250 mg/kg + 100 mg/kg)] and 34 mice were used for experiments under urethane anesthesia (pretreatment with Saline *n* = 9, Vehicle *n* = 6, CBD *n* = 6, NX *n* = 7, NX + CBD *n* = 6).

### Drugs and dosing

Drugs were administered as stock solutions except for CBD that was dissolved in a vehicle of 10% DMSO, 10% Tween-80, and 80% saline. Since CBD is not dilutable in saline, but naloxone is, saline controls were utilized for naloxone comparisons and vehicle controls for CBD and CBD + NX. CBD + NX was given together as a single i.p. injection. In our pilot experiments in mice low-dose CBD (10 mg/kg i.p.) pretreatment prevented respiratory depression from therapeutic doses of morphine, but lost efficacy as the morphine dose increased (Wiese, 2022). Thus, we based the dose studied here on the highest FDA approved CBD dose, 25 mg/kg for Epidiolex for Dravet syndrome (I. Jazz Pharmaceuticals, 2024), which is well tolerated in humans (Hurd et al., 2015; Lo et al., 2023; Manini et al., 2015; Mlost et al., 2020; Peng et al., 2022; Taylor et al., 2018). We calculated that this corresponds to a mouse equivalent dose of 250 mg/kg i.p. (Shen et al., 2019). We used a high-dose of fentanyl (McKesson; 50 mg/kg, i.p.) to ensure significant respiratory depression (Haouzi et al., 2021). We used 100 mg/kg Naloxone to assure its effectiveness, since a smaller dose attenuates, but does not fully prevent respiratory depression in mice evoked by a fentanyl dose smaller than the one used in this study (Varshneya et al., 2022). Pretreatment conditions were assigned in a quasi-random order, with mice from the same cage distributed across different pretreatment groups, and the experimenter was blinded to pretreatment identity during all testing.

### Whole-body plethysmography

**Figure 1:**
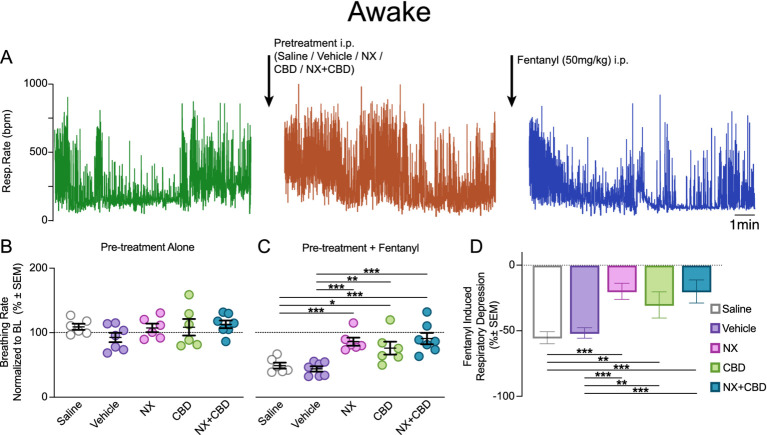
Pretreatments with CBD, NX or both mitigate fentanyl-induced respiratory depression in awake mice. **(A)** An example of respiratory recording in awake mice. After baseline recording in the whole-body plethysmograph, mice were taken out of plethysmograph to receive pretreatment i.p. injection (Saline, Vehicle, NX, CBD or NX + CBD), followed by 10 min of recording in the plethysmograph, and then taken out to receive fentanyl 50 mg/kg i.p. injection, and a final 15 min plethysmograph recording. **(B)** Breathing frequency in awake mice following pretreatment alone was not altered compared to within-subject baseline or across groups (all *p* > 0.05, see Supplementary Table 1). **(C)** Pretreatment with NX, CBD or CBD + NX significantly attenuated a decrease in breathing frequency evoked by fentanyl in awake mice compared with pretreatments with saline or vehicle (Supplementary Table 2). **(D)** Following fentanyl administration awake mice pretreated with saline or vehicle had a significantly depressed breathing frequency ~50%, while NX, CBD, or CBD + NX only reduced breathing frequency by 30% or less. *Post-hoc* Tukey comparison significant * - with *p* < 0.05, ** - with *p* < 0.01, *** - with *p* < 0.001. See Supplementary Tables for statistics results.

Breathing frequency in awake and freely moving mice was assessed using whole-body plethysmograph (Buxco Research Systems). The plethysmograph (v ~ 0.48 L, air flow ~ 1 L/min) was connected to a pressure transducer (model DP103-10-871, Validyne Engineering) and a carrier demodulator (model CD15-A-2-A-1, Validyne Engineering). The recordings consisted of 1 hour acclimation period/baseline, 10 min after administration of CBD, NX, saline, vehicle, or CBD + NX i.p.; and 10 min after fentanyl administration (Figure 1A). All drugs were diluted in either saline or vehicle to be administered to each mouse with an injection volume of 10 mL/kg.

### ICV infusion preparation and OIPA model

**Figure 2:**
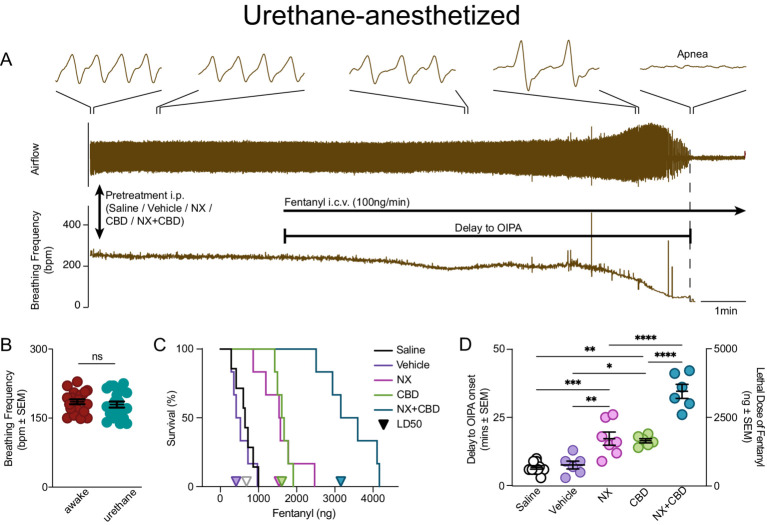
Pretreatments with CBD, NX or both delay fentanyl-induced OIPA in urethane-anesthetized mice. **(A)** An example of respiratory recording in urethane-anesthetized mice. Urethane-anesthetized mice received pretreatment i.p. injection (Saline, Vehicle, NX, CBD, or NX + CBD), followed by a continuous Fentanyl i.c.v. infusion (100 ng/min) into the IV ventricle until persistent apnea, i.e., irreversible cessation of breathing for longer than 1 min. Delay to OIPA was calculated from the start of fentanyl infusion until the start of terminal persistent apnea. **(B)** Average breaths per minute (BPM) in urethane-anesthetized mice (n = 20) were the same as in awake mice (*n* = 20), *p* = 0.48. **(C)** Survival curves of urethane anesthetized mice during continuous ICV fentanyl infusion were used to determine the LD_50_ of fentanyl after pretreatment (triangles correspond to LD_50_ values) with saline (625 ng) and vehicle (413 ng). CBD (1,608 ng) or NX (1,536 ng) pretreatment significantly increased survival (saline vs. NX: *p* = 0.001, vehicle vs. CBD *p* < 0.001, Supplementary Table 4) and increased the LD_50_ of fentanyl. Pretreatment with CBD + NX significantly increased survival further (CBD + NX vs. CBD, NX, saline and vehicle: all *p* < 0.001, Supplementary Table 4) and increased the LD_50_ of fentanyl (3,155 ng). **(D)** Delay to OIPA onset from the start of continuous fentanyl infusion following pretreatment with saline or vehicle in urethane anesthetized mice induced a persistent apnea was ~9 min; time to OIPA onset was significantly increased after pretreatment with CBD or NX (~17 min each); time to OIPA onset was further increased following pretreatment with CBD + NX (~35 min; Supplementary Table 3). *Post-hoc* Tukey comparison significant * - with *p* < 0.05, ** - with *p* < 0.01, *** - with *p* < 0.001. See Supplementary Tables for statistics results.

Isoflurane anesthetized mice were transitioned to urethane (1000–1,500 mg/kg, i.p.), with body temperature maintained by a heating pad and airflow signal continuously recorded. The dorsal side of the skull was exposed, a small hole was drilled (A/P-4.9, M/L 0.0) and the tip of a Hamilton syringe was lowered into the IVth ventricle (D/V-2.25). CBD (250 mg/kg), naloxone (NX; 100 mg/kg), saline, vehicle, or CBD + NX (CBD + NX; 250 mg/kg + 100 mg/kg), i.p., was given 10 min prior to the start of a continuous fentanyl infusion. Fentanyl (50mcg/mL) was infused at a rate of 100 ng/min until onset of OIPA. We calculated time to OIPA onset and lethal fentanyl dose, which we defined as the dose of fentanyl when all breathing activity stopped, resulting in a fatal persistent apnea, i.e., cessation of breathing for longer than 1 min (Figure 2A). We chose i.c.v. infusion over i.v. or i.p. infusion as this route of fentanyl administration evokes an almost immediate response, greatly reducing inter-individual variability and allows more precise estimation of lethal fentanyl dose.

### Statistics and reproducibility

Airflow signals were acquired, amplified, digitized, stored, and analyzed using LabChart 8 software and PowerLab 8/16 data acquisition system (ADInstruments). Parameters were calculated offline from the airflow signal. Prism (GraphPad Software) and IGOR software (WaveMetrics) were used to perform analysis. ANOVA analyses were used for within-subject and across treatment groups. Tukey *post hoc* tests were used when significant differences between groups were found. Survival curves were created using Prism and compared using Logrank Kaplan–Meier analysis with post-hoc pairwise comparisons adjusted using 2-stage method of Benjami, Krieger and Yekutielu False-Discovery Rate. Significance was set at *p* ≤ 0.05 for all comparisons.

## Results

### Awake behaving mice

In awake unrestrained mice, we determined the effect of a single fentanyl dose (50 mg/kg i.p.) that was preceded by i.p. saline, vehicle, NX (100 mg/kg), CBD (250 mg/kg), or CBD + NX (Figure 1A). Saline control was used for comparison with naloxone and vehicle control for comparison with CBD and CBD + NX (see METHODS). There was no impact on breathing frequency following pretreatment injections across groups compared to the within-subject baseline (*p* = 0.56, Figure 1B, Supplementary Table 1). After pretreatment with saline or vehicle, fentanyl significantly decreased breathing frequency by 54.9 ± 4.50% and 51.4 ± 5.82%, respectively, (both *p* < 0.001, Figure 1C, Supplementary Table 2). In contrast, after pretreatment with CBD, NX and CBD + NX fentanyl significantly reduced breathing frequency by 30.0 ± 2.67%, 19.6 ± 3.16% and 19.8 ± 5.34%, respectively, (*p* < 0.001, *p* = 0.002 and *p* < 0.001 respectively; Figure 1D; Supplementary Table 2), yet this reduction was significantly attenuated compared to vehicle or saline pretreatment groups (*p* = 0.003, *p* = 0.001, and *p* < 0.001; Figure 1D, Supplementary Table 2). Breathing frequency after fentanyl was not significantly different in CBD, NX, and CBD + NX pretreatment groups (*p* = 0.339, *p* = 0.148 and *p* = 0.636, Figure 1D, Supplementary Table 2). To determine if these pretreatments decrease the lethality of fentanyl, we next examined their effects on breathing during continuous fentanyl infusion in anesthetized mice. See Supplementary Table 1 for repeated-measures ANOVA results and Supplementary Table 2 for detailed mixed 2 × 5 (timepoint × pretreatment) ANOVA results.

### Urethane anesthetized mice

Opioids induce hyperactivity, e.g., increased locomotor activity, in awake mice (Varshneya et al., 2021), and this effect can contribute to the fact that awake mice tolerate relatively high doses of opioids compared to humans. We chose urethane anesthesia to investigate the fentanyl LD_50_ as opioids can induce persistent apnea, i.e., OIPA, in mice under urethane anesthesia (Figure 2A), unlike isoflurane or ketamine, and it preserves awake breathing frequency in mice (Figure 2B). Here, urethane anesthetized mice, following i.p. pretreatment with saline, vehicle, NX (100 mg/kg), CBD (250 mg/kg), or CBD + NX, received a continuous i.c.v. infusion of fentanyl (100 ng/min; Figure 2A). We then determined the time during continuous fentanyl infusion until OIPA onset and the corresponding dose of fentanyl, as well as the median lethal dose (LD_50_) from survival functions for each pretreatment (see METHODS). Following pretreatment with saline, the LD_50_ of fentanyl was 625 ng, and with the vehicle was 413 ng (Figure 2C), with the onset of OIPA after 6.46 ± 0.93 and 5.45 ± 1.06 min, respectively, (Figure 2D). Following pretreatment with NX or CBD there was a significant increase in the delay to OIPA onset to 15.7 ± 2.24 and 16.5 ± 0.73 min, respectively, (Figure 2D; saline vs. NX: *p* < 0.001, vehicle vs. CBD: *p* = 0.010; Supplementary Table 3) and an increase in fentanyl LD_50_ (CBD LD_50_ = 1,608 ng, NX LD_50_ = 1,536 ng; Figure 2D). Pretreatment with NX or with CBD significantly increased survival curves compared with saline and vehicle pretreatments (Figure 2C; saline vs. NX: *p* = 0.001, vehicle vs. CBD *p* < 0.001, Supplementary Table 4) Furthermore, compared to CBD or NX alone, pretreatment with CBD + NX significantly increased to delay to OIPA onset to 34.1 ± 2.70 min (Figure 2D; CBD + NX vs. CBD, NX, saline and vehicle: all *p* < 0.001; Supplementary Table 3), significantly increased survival (Figure 2C; CBD + NX vs. CBD, NX, saline and vehicle: all *p* < 0.001, Supplementary Table 4) and increased the LD_50_ of fentanyl (3,155 ng; Figure 2C). See Supplementary Table 3 for detailed ANOVA results and Supplementary Table 4 for detailed Logrank comparisons of survival curves of various pretreatment groups.

## Discussion

In awake mice, CBD pretreatment was as effective as NX pretreatment at mitigating fentanyl-induced depression of breathing frequency. In anesthetized mice during a continuous fentanyl infusion, CBD pretreatment significantly increased delay to OIPA and survival and also increased the LD_50_ of fentanyl compared to vehicle pretreatment. These effects were comparable to those of mice receiving high dose NX. Furthermore, CBD + NX pretreatment was more efficacious than either CBD or NX at increasing both delay to OIPA and the LD_50_.

While fentanyl depresses breathing in awake behaving mice, even very high doses do not evoke a persistent apnea or result in loss of consciousness as seen in humans [e.g., see Figure 3H in Bachmutsky et al. (2020)] for the respiratory signal of a mouse after 4.5 mg injection of fentanyl, an amount considered twice the lethal dose in humans. Yet, a modest decrease in ventilation in rodents, e.g., 30–60% decrease in breathing frequency, is generally considered representative of OIPA in humans. This assumes that mechanisms slowing breathing and evoking a persistent apnea are the same. Yet, there is no data confirming that this is the case. Breathing persists for many manipulations that depress breathing, e.g., sleep, vagotomy. Here, i.c.v. fentanyl infusion reliably and consistently evoked persistent apnea in urethane-anesthetized mice (Figure 2A). This mode of fentanyl administration was chosen due to the rapidity of the respiratory response, precise control of the infused dose and reduced variability. Large doses of opioids, such as those that precipitate an overdose, result in loss of consciousness in humans, with breathing in a state similar to that during anesthesia. Urethane anesthesia, in comparison to other anesthetics, preserves the baseline excitability level of breathing central pattern generator components and is suggested to mimic sleep (Pagliardini et al., 2013). We suggest that the urethane-anesthetized mice model used here resembles an opioid overdose in humans, thus suited to investigate OIPA. Here, we demonstrate the effectiveness of CBD in both awake mice, where it significantly reduced OIRD induced by i.p. injection of fentanyl, and in urethane-anesthetized mice, where it significantly increased i.c.v. dose of fentanyl required to induce OIPA.

The mechanisms by which high-dose CBD antagonizes respiratory depression and OIPA are currently not known. With more than 72 known protein targets, including serotonin, opioid, and dopamine receptors, as well as TRPV channels (Britch et al., 2021; Manini et al., 2015; Mlost et al., 2020; Peng et al., 2022; Bosquez-Berger et al., 2023; Kathmann et al., 2006), and an ability to block adenosine transport (Iffland and Grotenhermen, 2017; Manini et al., 2015; Mlost et al., 2020; Sideris and Doan, 2024; Peng et al., 2022), the scope of potential mechanisms at any site where CBD can modulate breathing (Del Negro et al., 2018; Ashhad and Feldman, 2020; Janczewski and Feldman, 2006) is legion. Among the possibilities: (i) opioids depress breathing, at least in part, by increasing GABAergic inhibition. CBD could antagonize the respiratory depressive effects of *μ*-opioid receptor activation in the breathing central pattern generator, including in the preBötzinger Complex, by modulating GABAergic and glutamate transmission, reducing inhibitory signals that lead to respiratory depression (Del Negro et al., 2018; Ashhad and Feldman, 2020; Janczewski and Feldman, 2006). (ii) CBD enhances 5-HT_1A_ receptor activity, which could increase serotonin signaling modulating breathing (Wiese et al., 2023). (iii) CBD acts as a negative allosteric modulator at μ-opioid receptors that could attenuate opioid-induced respiratory depression, yet preserve analgesia (Kang et al., 2022; Manini et al., 2015; Peng et al., 2022; Kathmann et al., 2006; Wiese et al., 2023). (iv) Since CBD did not affect breathing frequency, its effect may only be manifest when breathing is (significantly) depressed, as in OIPA but not in OIRD. Investigating these various possibilities is beyond the scope of this paper.

The protocol here is markedly different when compared to the effect of (near) complete block of all μ-opioid receptors following the onset of symptoms of an overdose, which is how NX is therapeutically used. After an acute CBD pretreatment prior to the administration of fentanyl in mice, 2–3 times more fentanyl was required to evoke OIPA. While we did not assess if analgesic or euphoric effects of fentanyl were preserved in mice following CBD administration, it has previously been established that CBD does not reduce opioid-induced analgesia in humans (Manini et al., 2015), while in mice it increased opioid-induced analgesia (Rodríguez-Muñoz et al., 2018). There is also substantial evidence that CBD decreases opioid craving both in humans (Hurd et al., 2019) and animals (Ren et al., 2009; de Carvalho and Takahashi, 2017). Although no study to date investigated whether CBD affects opioid-induced euphoria, it was shown that CBD does not induce euphoria and does not interfere with euphoria induced by THC, a psychoactive cannabinoid (Pennypacker and Romero-Sandoval, 2020). We suggest that in humans CBD administration prior to opioid use can decrease the incidence of OIPA and fatality without significantly affecting analgesia, euphoria, or precipitating acute withdrawal. This does not preclude the use of NX as a rescue therapeutic, but may reduce the need for NX, expand the therapeutic window when NX can be administered, and/or reduce NX dose required for resuscitation. Importantly, CBD administration does not preclude NX effectiveness, evident in the cumulative effect of CBD + NX.

CBD is tolerable in mice (Iffland and Grotenhermen, 2017; Zanelati et al., 2010; Crivelaro do Nascimento et al., 2020; Deiana et al., 2012; Elsaid et al., 2019; Stella, 2023). Low dose CBD (10 mg/kg i.p.) mitigates morphine-induced respiratory depression resulting from therapeutic doses of morphine (10 mg/kg i.p.), but the effect is lost as the morphine dose increases to 30 mg/kg (Wiese et al., 2021; Wiese, 2022). In humans, CBD doses equivalent to 250 mg/kg in mice used in the current study are well tolerated, e.g., up to 6,000 mg oral solution (Taylor et al., 2018) and 200–800 mg acute dosing for people who use drugs (Hurd et al., 2015; Lo et al., 2023; Hurd et al., 2019). In a mouse model of Dravet syndrome, 10-50 mg/kg i.p. CBD was ineffective against seizure frequency, but was effective at 100-200 mg/kg i.p. doses (Kaplan et al., 2017), similar to the dose used in this study. Since Epidiolex, an oral CBD medication for Dravet syndrome, reduces seizure frequency in humans at FDA-approved dose up to 25 mg/kg, a dosing regimen similar to Epidiolex might also be effective in humans against OIRD/OIPA. As mice require substantially higher doses of fentanyl than humans or large primates to evoke respiratory depression or OIPA, the CBD dosing and route of administration necessary to achieve its preventative effect in humans would need to be determined in follow-up clinical studies.

The ultimate utility of this work is contingent on whether it applies to humans. Given that high dose CBD: (i) is FDA approved (Mlost et al., 2020); (ii) is well tolerated in humans (Taylor et al., 2018) including when given concurrently with intravenous fentanyl administration (Manini et al., 2015); (iii) does not counteract opioid analgesic or euphoric effects (Manini et al., 2015), (iv) potentiates the effects of naloxone (Kang et al., 2022; Bosquez-Berger et al., 2023), and (v) is available without a prescription in the US, there are few barriers to determine if CBD is equally as effective against OIPA in humans as it is mice. This proof of concept using CBD as a prophylactic therapeutic for prevention of fatal OIPA in mice has considerable potential for public health benefit.

## Data Availability

The raw data supporting the conclusions of this article are available at Harvard Dataverse (https://doi.org/10.7910/DVN/KDZ0I0).
