# Supplementary material for: Proof of concept for high-dose Cannabidiol pretreatment to antagonize opioid induced persistent apnea in mice
Source: Front Neurosci. 2025 Oct 8;19:1654787. doi: 10.3389/fnins.2025.1654787 (PMC12540426; doi:10.3389/fnins.2025.1654787)
Supplement: Supplementary file 1 [file Table_1.docx]

**Supplementary Table 1**

*ANOVA Summary of “Breathing Frequency” variable in Awake mice*

*Repeated-Measures ANOVA: baseline, after pretreatment, after fentanyl*

Effects P Value F (DFn, DFd)

Timepoint <0.001*** F (2, 81) = 61.07

Within-Subjects Comparisons

Tukey Multiple Comparisons Test Adjusted P Value

Baseline vs. After pretreatment 0.556

Baseline vs. After fentanyl <0.001***

After pretreatment vs. After fentanyl <0.001***
