# Supplementary material for: Proof of concept for high-dose Cannabidiol pretreatment to antagonize opioid induced persistent apnea in mice
Source: Front Neurosci. 2025 Oct 8;19:1654787. doi: 10.3389/fnins.2025.1654787 (PMC12540426; doi:10.3389/fnins.2025.1654787)
Supplement: Supplementary file 2 [file Table_2.docx]

**Supplementary Table 2**

*ANOVA Summary of “Breathing Frequency Normalized to BL” variable in Awake mice*

*2x5 Mixed ANOVA: Timepoint (within-subjects: after pretreatment vs. after fentanyl) X Pretreatment (between-subjects: Saline, CBD, BX, Vehicle, NX+CBD)*

Effects P Value F (DFn, DFd)

Timepoint <0.001*** F (1, 31) = 194.4

Pretreatment 0.008** F (4, 27) = 4.254

Timepoint x Pretreatment <0.001*** F (4, 27) = 8.250

Between-Subjects Comparisons

Tukey Multiple Comparisons Test Adjusted P Value

After Pretreatment i.p.

Saline vs. Vehicle 0.149

Saline vs. CBD 0.957

Saline vs. NX 0.888

Saline vs. NX+CBD 0.725

Vehicle vs. CBD 0.165

Vehicle vs. NX 0.192

Vehicle vs. NX+CBD 0.066

CBD vs. NX 0.931

CBD vs. NX+CBD 0.683

NX vs. NX+CBD 0.619

After Fentanyl i.p.

Saline vs. Vehicle 0.614

Saline vs. CBD 0.014*

Saline vs. NX <0.001***

Saline vs. NX+CBD <0.001***

Vehicle vs. CBD 0.003**

Vehicle vs. NX <0.001***

Vehicle vs. NX+CBD <0.001***

CBD vs. NX 0.339

CBD vs. NX+CBD 0.148

NX vs. NX+CBD 0.636

Within-Subjects Comparisons

Saline pretreatment group

After Pretreatment vs. After fentanyl <0.001***

Vehicle pretreatment group

After Pretreatment vs. After fentanyl <0.001***

CBD pretreatment group

After Pretreatment vs. After fentanyl <0.001***

NX pretreatment group

After Pretreatment vs. After fentanyl 0.002**

CBD+NX pretreatment group

After Pretreatment vs. After fentanyl <0.001***
