# Supplementary material for: Proof of concept for high-dose Cannabidiol pretreatment to antagonize opioid induced persistent apnea in mice
Source: Front Neurosci. 2025 Oct 8;19:1654787. doi: 10.3389/fnins.2025.1654787 (PMC12540426; doi:10.3389/fnins.2025.1654787)
Supplement: Supplementary file 3 [file Table_3.docx]

**Supplementary Table 3**

*ANOVA Summary of “Delay to OIPA” variable in Urethane-anesthetized mice*

*Between-Subjects ANOVA: Pretreatment (Saline, CBD, BX, Vehicle, NX+CBD)*

Effects P Value F (DFn, DFd)

Pretreatment <0.001*** F (4, 30) = 44.83

Between-Subjects Comparisons

Tukey Multiple Comparisons Test Adjusted P Value

Saline vs. Vehicle 0.995

Saline vs. CBD 0.001**

Saline vs. NX <0.001***

Saline vs. NX+CBD <0.001***

Vehicle vs. CBD 0.010*

Vehicle vs. NX 0.003**

Vehicle vs. NX+CBD <0.001***

NX vs. CBD 0.997

NX vs. NX+CBD <0.001***

CBD vs. NX+CBD <0.001***
