# Supplementary material for: Proof of concept for high-dose Cannabidiol pretreatment to antagonize opioid induced persistent apnea in mice
Source: Front Neurosci. 2025 Oct 8;19:1654787. doi: 10.3389/fnins.2025.1654787 (PMC12540426; doi:10.3389/fnins.2025.1654787)
Supplement: Supplementary file 4 [file Table_4.docx]

**Supplementary Table 4**

*Logrank (Kaplan-Meier) analysis of survival curves between different pretreatments*

Survival df χ^2^ P-value

Pretreatment 4 45.25 <0.001***

Pairwise Comparisons

χ^2^ FDR-Adjusted P-value

Saline vs. Vehicle 0.5017 0.4787

Saline vs. CBD 12.64 <0.001***

Saline vs. NX 10.71 0.001**

Saline vs. NX+CBD 12.64 <0.001***

Vehicle vs. CBD 12.09 <0.001***

Vehicle vs. NX 9.994 0.001**

Vehicle vs. NX+CBD 12.09 <0.001***

NX vs. CBD 0.006108 0.9377

NX vs. NX+CBD 12.09 <0.001***

CBD vs. NX+CBD 12.09 <0.001***
